# Supplementary material for: Delayed and Interrupted Ventilation with Excess Suctioning after Helping Babies Breathe with Congolese Birth Attendants
Source: Children (Basel). 2023 Mar 30;10(4):652. doi: 10.3390/children10040652 (PMC10137041; doi:10.3390/children10040652)
Supplement: Supplementary file 1 [file children-10-00652-s001.zip › children-2257167-supplementary.pdf]

Supplemental Table S1. Definitions for key actions and additional notes

Action	Definition	Additional Notes
Skin-to-skin	Direct contact of the newborn with the mother's abdomen or chest	If a blanket was between the newborn and the mother, this was not counted as skin-to-skin
Dry/stimulate	Wiping the newborn with a blanket or towel, or prompting the newborn to breathe by such actions as rubbing the back or flicking the foot	Logged for any occurrence of either drying or stimulation
Suction	Using a suction bulb or suction catheter to remove secretions from the mouth or nose	Start time of suctioning was when bulb or catheter entered the mouth or nose
Ventilate	Delivering breaths to a newborn via a bag and mask	Start time of ventilation was when first breath was delivered (i.e., not the timing of the mask being applied to the face)
Cord clamp	When the provider uses a clamp or tie to stop blood flow through the cord	This was not the time of the cord being cut
Improve ventilation	One or more of the following actions: reapplying the mask, repositioning the head, opening the mouth slightly or squeezing the bag harder	Logged all episodes of clearing the mouth or nose of secretions as 'suctioning' rather than 'improve ventilation'

Supplemental Table S2. Order of resuscitation practices by breathing status at 30 s after birth

**Order of Resuscitation Care Practices**	**Breathing well by 30 s after Birth *n* = 1818**		**Not Breathing well by 30 s after Birth *n* = 774**	
	Resuscitations during which both actions occurred, *n* (%)	Frequency, *n* (%)	Resuscitations during which both actions occurred, *n* (%)	Frequency, *n* (%)
Skin-to-skin before				
Dry/stim	1633 (89.8)	1125 (68.9)	681 (88.0)	450 (66.1)
Suction	911 (50.1)	901 (98.9)	530 (68.5)	509 (96.0)
Ventilation	7 (0.4)	7 (100)	44 (5.7)	44 (100)
Cord clamp	1605 (88.3)	1597 (99.5)	660 (85.3)	656 (99.4)
Dry/stim before				
Suction	1029 (56.6)	1017 (98.8)	608 (78.6)	590 (97.0)
Ventilation	8 (0.4)	8 (100)	73 (9.4)	73 (100)
Cord clamp	1783 (98.1)	1760 (98.7)	751 (97.0)	733 (97.6)
Suction before				
Ventilation	8 (0.4)	8 (100)	73 (9.4)	73 (100)
Cord clamp	1010 (55.6)	494 (48.9)	589 (76.1)	400 (67.9)
Cord clamp before				
Ventilation	8 (0.4)	8 (100)	69 (8.9)	69 (100)
